# Impact of *Aspergillus flavus* Infection on the Rhizosphere Bacterial Microbiota of Peanut (*Arachis hypogaea* L.)

**DOI:** 10.3390/toxins18030131

**Published:** 2026-03-05

**Authors:** Qiujun Lin, Xianxin Wu, Lina Li, Tianshu Peng, Xun Zou, Guang Li, Jianzhong Wang, Xiaoqian Tang, Xiaofeng Yue, Chunjing Guo, Peiwu Li

**Affiliations:** 1 Institute of Agricultural Quality Standards and Testing Technology, Liaoning Academy of Agricultural Sciences, Shenyang 110161, China; linqiujun85@163.com (Q.L.); wuxianxin1225@163.com (X.W.); lln231118@163.com (L.L.); tspenglaas@163.com (T.P.); zouxun90@163.com (X.Z.); lngtgh@163.com (G.L.); zbswjz72@163.com (J.W.); 2 Institute of Oil Crops, Chinese Academy of Agricultural Sciences, Wuhan 430062, China; tangxiaoqian@caas.cn (X.T.); yuexf2017@caas.com (X.Y.); 3 Xianghu Laboratory, Hangzhou 311215, China

**Keywords:** *Aspergillus flavus*, peanut, rhizosphere bacterial microbiome, bacterial diversity, bacterial community changes, plant-microbe interaction

## Abstract

This study investigated the effects of inoculating peanuts with two *Aspergillus flavus* strains (*Aspergillus flavus* CGMCC 3.4408 and *A. flavus* LNZW 23) on plant growth and the rhizosphere bacterial community. Infection significantly inhibited peanut growth. By 60 days post-inoculation (dpi), plant height in inoculated groups (CGMCC 3.4408, 26.4 cm; LNZW 23, 25.5 cm) was significantly lower than in the non-inoculated control (CK, 32.3 cm), with concomitant significant reductions in shoot and root biomass. Analysis of rhizosphere microbiota revealed that early infection (7 dpi) reduced bacterial species richness and phylogenetic diversity. Beta diversity analysis (PCoA) confirmed a significant divergence in microbial community structure between inoculated and control groups over time, with a statistically significant difference also observed between the two inoculated strains (*p* = 0.016). In terms of community composition, Proteobacteria, Acidobacteriota, and Actinobacteria were the three dominant phyla. At the genus level, infection altered the relative abundance of key taxa; genera such as *KD4-96*, *Vicinamibacteraceae*, and *RB41* decreased at 7 dpi, while *Sphingomonas* remained relatively stable. By 60 dpi, community dominance increased, marked by rising abundances of Actinobacteria and Proteobacteria. In conclusion, *A. flavus* infection not only suppresses peanut growth but also persistently alters its rhizosphere microbial community, with effects demonstrating both time-dependency and strain-specificity.

## 1. Introduction

Peanut (*Arachis hypogaea* L.) is a globally widespread and crucial oilseed and economic crop, holding significant importance in agricultural production and food security [1,2]. Its growth cycle is regulated by multiple factors, including variety, climate, soil, and cultivation management. Peanut kernels are rich in oils, proteins, vitamins, and minerals, serving not only as a vital dietary component but also being extensively used in food processing, the oil industry, and feed production. According to the Food and Agriculture Organization (FAO) of the United Nations, the global cultivated area for peanuts reached approximately 31 million hectares with a total production of about 54 million tons in 2022, highlighting its significant role in alleviating vegetable oil and protein demands and supporting rural economies [3].

However, peanuts are highly susceptible to infection by *Aspergillus flavus* during field growth, harvest, and storage. *A. flavus* is a common saprophytic fungus widely distributed in soil, air, and plant residues, capable of rapid proliferation and infection of peanut pods under suitable temperature and humidity conditions. This fungus not only causes yield losses but, more critically, produces highly toxic aflatoxins. Among these, aflatoxin B1 (AFB1) is classified as a Group I human carcinogen by the International Agency for Research on Cancer [4]. Long-term dietary exposure to AFB1 is significantly associated with an increased risk of hepatocellular carcinoma, posing a serious public health burden, particularly in parts of Africa and Asia [5]. Global annual economic losses in agriculture and health due to aflatoxin contamination amount to tens of billions of US dollars [6].

The root system functions as a critical interface for plant–soil interactions, and the rhizosphere microbiome is the core microecosystem governing peanut growth, nutrient acquisition, and resistance to biotic stress [7,8,9]. For peanuts—a legume highly dependent on rhizosphere symbiosis—a stable microbial community is indispensable to agronomic performance. It facilitates the mobilization of soil nitrogen, phosphorus, and potassium for plant uptake [10], synthesizes growth-promoting metabolites such as indole-3-acetic acid and cytokinins [11], and primes systemic resistance against major soil-borne pathogens, including *Fusarium oxysporum* f. sp. *arachidis* (*fusarium* wilt), *Pythium aphanidermatum* (root rot), and *A. flavus* (pod rot and aflatoxin contamination) [12,13,14]. The disruption of this microbial equilibrium can lead to substantial yield penalties and significant deterioration of kernel quality [15]. For instance, disturbances to rhizosphere bacterial communities have been associated with yield reductions ranging from 10% to 93% under different cropping systems [16], while shifts in specific beneficial taxa such as *Bradyrhizobium* and *Streptomyces* can impair soil nutrient cycling and subsequently affect peanut productivity [17]. Furthermore, continuous cropping practices that destabilize the microbial balance have been shown to decrease enzyme activities (e.g., L-leucine aminopeptidase) and increase the abundance of fungal pathogens, directly contributing to yield decline [18]. These microbial perturbations not only reduce pod yield but also compromise kernel quality by altering key metabolic pathways and nutrient accumulation [19].

*A. flavus* infection induces profound rhizosphere perturbations in peanut, including reduced microbial diversity, altered community structure, and repressed expression of plant defense- and nutrient cycling-related functional genes [20]. Beyond these rhizosphere-level effects, the pathogen exerts direct pathogenic damage through pod rot, seed deterioration, and substantial yield losses under severe infection conditions [21,22]. A major concern is the contamination with Group I carcinogenic aflatoxins—including AFB_1_, AFB_2_, AFG_1_, and AFG_2_—which pose serious food safety risks [21]. Additionally, *A. flavus* produces cell wall-degrading enzymes and mycotoxins that inhibit seed germination and seedling establishment, further compromising crop productivity [22]. Most relevant studies to date have focused on direct plant-fungus interactions, with limited attention to rhizosphere microbiome-mediated indirect effects [22,23]. Given that bacteria account for the majority of rhizosphere microbial biomass and possess exceptional metabolic diversity, they represent a critical yet understudied component in elucidating the indirect pathogenic mechanisms of *A. flavus* [20,23].

While *A. flavus* is known to cause direct pathological damage through pod rot and aflatoxin contamination [21,22], accumulating evidence suggests that its pathogenic success may also involve the manipulation of the rhizosphere microbiome [20,23]. However, current understanding of the tripartite interaction among *A. flavus*, peanut, and the rhizosphere microbiome remains fundamentally incomplete [24,25]. Three critical knowledge gaps persist: (i) the absence of systematic quantification of dynamic rhizosphere bacterial community shifts during both the early colonization and late pathogenic stages of infection, despite bacteria being the most rapid responders to environmental perturbations; (ii) unknown comparative effects of toxigenic versus non-toxigenic *A. flavus* strains on rhizosphere bacterial assemblages—a critical comparison given their natural co-existence and potential to exert contrasting selective pressures; and (iii) a lack of causal evidence directly linking bacterial dysbiosis to peanut growth inhibition and yield suppression within the context of *A. flavus* pathogenesis.

To address these gaps, this study focuses on the rhizosphere bacterial community as an entry point for deciphering the indirect mechanisms of *A. flavus* pathogenicity. Specifically, we aim to: (1) assess the temporal effects of *A. flavus* infection on peanut growth parameters at 7 and 60 days post-inoculation; (2) characterize the dynamic changes in rhizosphere bacterial diversity and community composition in response to infection; (3) compare the strain-specific effects of two *A. flavus* isolates (CGMCC 3.4408 and LNZW 23) on the rhizosphere microbiome; and (4) elucidate the mechanistic links between shifts in the bacterial community and the suppression of peanut growth. By establishing a foundational understanding of the bacterial response, this study will provide a theoretical framework for the development of microbiome-based biocontrol strategies [8,26]. We acknowledge that a holistic understanding of the rhizosphere ecosystem will ultimately require integration of fungal community dynamics, which will be the focus of our subsequent investigations.

## 2. Results

### 2.1. Impact of A. flavus Infection on Peanut Growth

**Figure 1:**
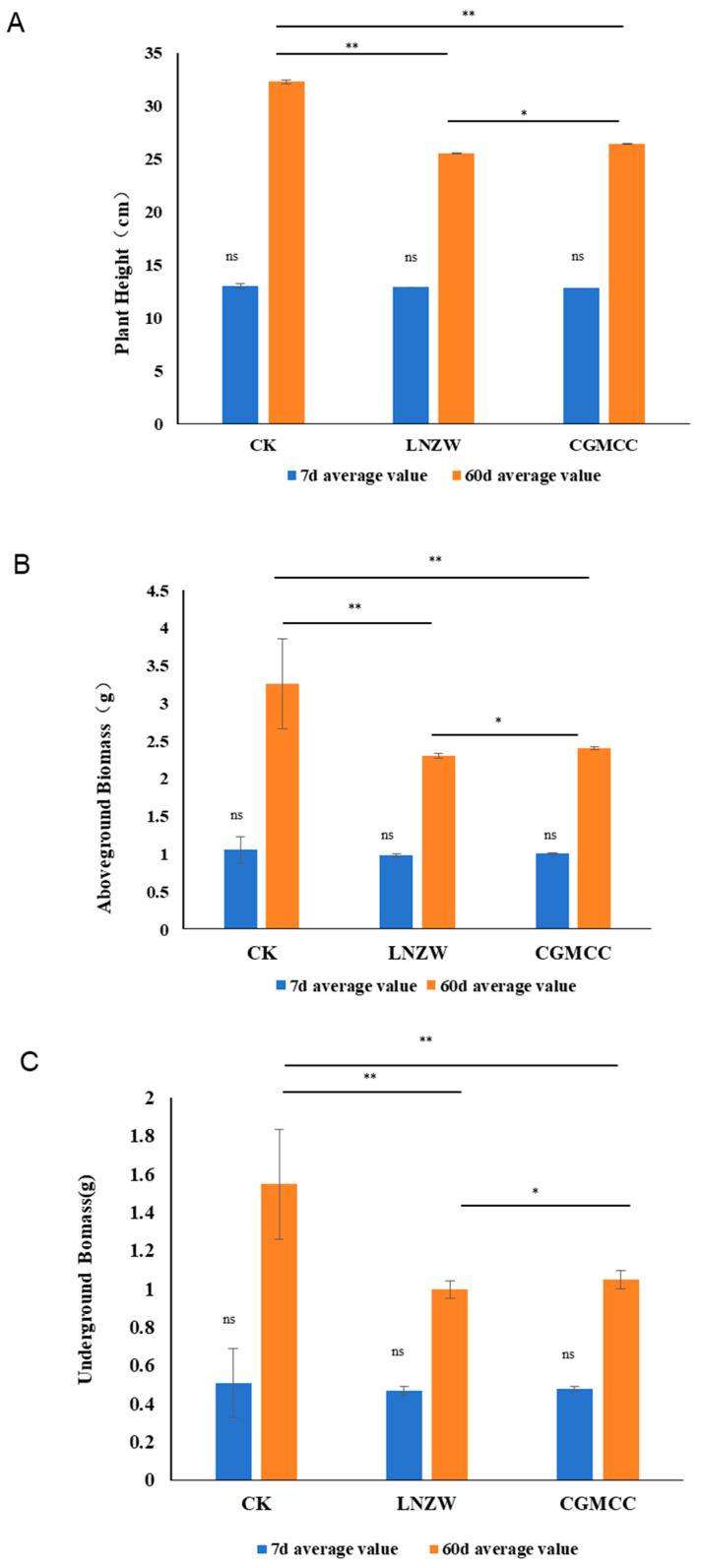
Differences in growth indicators of peanut plant height (**A**), aboveground biomass (**B**), and underground biomass (**C**) between 7 and 60 days after inoculation with *A. flavus*. Note: ** *p* < 0.01 (Significant), * *p* < 0.05 (Moderately significant), ns, No significant.

Measurements of plant height, shoot biomass, and root biomass during the experimental period are presented in Figure 1. At 7 days post-inoculation (dpi), there was no significant difference in plant height between the infected groups and the control group. From 60 dpi onwards, height growth in the infected groups was significantly inhibited. The average plant height in the control group reached 32.3 cm, while it was 26.4 cm in the CGMCC group and only 25.5 cm in the LNZW group (Supplementary Materials Table S1). Biomass measurements at 60 dpi showed that both shoot (Figure 1B) and root biomass (Figure 1C) in the infected groups were significantly lower than those in the control group.

### 2.2. Changes in the Rhizosphere Microbial Diversity of Peanut

#### 2.2.1. Alpha Diversity Analysis

**Figure 2:**
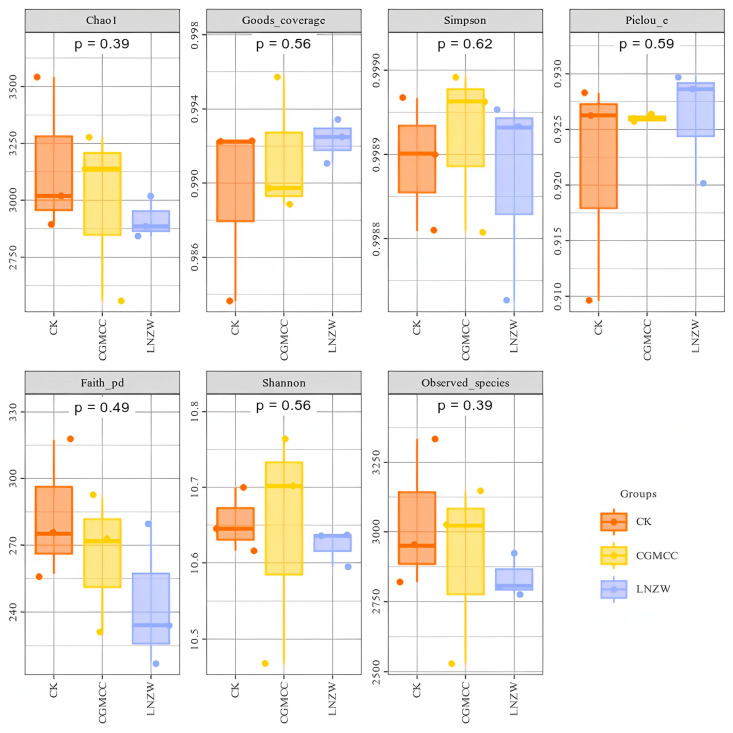
Bacterial alpha diversity indices at 7 days post-inoculation with *A. flavus.* The numbers above each panel are the *p*-values derived from Kruskal-Wallis tests, used to determine significant differences between the two groups for each alpha diversity measure (*p*-value < 0.05 was considered statistically significant).

**Figure 3:**
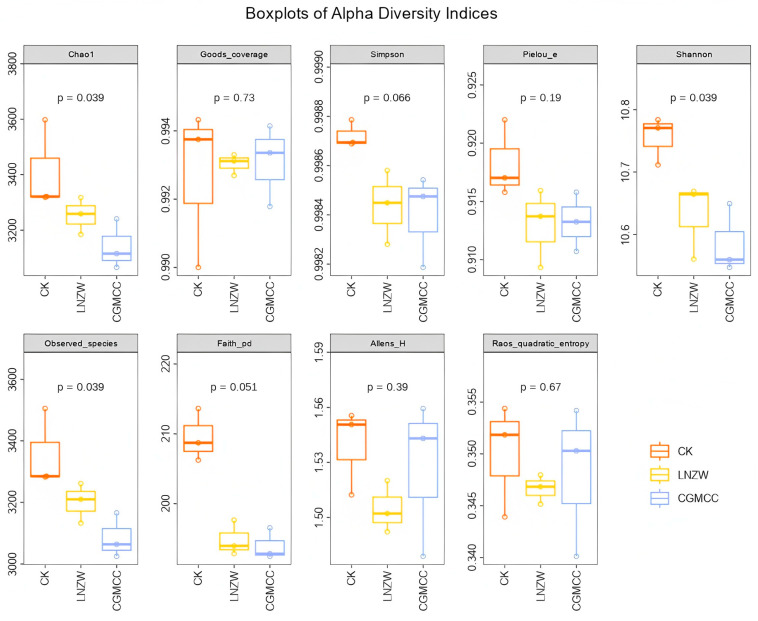
Bacterial alpha diversity indices at 60 days post-inoculation with *A. flavus*. The numbers above each panel are the *p*-values derived from Kruskal-Wallis tests, used to determine significant differences between the two groups for each alpha diversity measure (*p*-value < 0.05 was considered statistically significant).

Alpha diversity of the bacterial communities in the peanut rhizosphere at 7 and 60 days post-inoculation with *A. flavus* was assessed using indices including Shannon, Simpson, Ace, and Chao1 (Tables S2 and S3; Figure 2 and Figure 3). The coverage for all samples exceeded 0.98, indicating sufficient sequencing depth and reliable data.

Simpson indices close to 1 indicated pronounced dominant species and relatively high evenness within communities. Shannon indices generally greater than 10.5 reflected a high overall level of diversity. At 7 dpi, the control group (CK) exhibited the highest species richness (based on Chao1 and Observed_species), followed by the CGMCC and LNZW groups, suggesting that *A. flavus* infection reduced species richness in the early stage. The phylogenetic diversity index (Faith_pd) was highest in the CK group and lowest in the LNZW group, indicating that inoculation affected the phylogenetic structure of the bacterial community.

At 60 dpi, the CK group maintained the highest richness, while the LNZW group showed slightly higher values than the CGMCC group, hinting at a potential recovery capacity in the LNZW treatment at later stages. Shannon indices ranked as CK > LNZW > CGMCC, with the CGMCC group showing the lowest diversity. Faith_pd remained significantly higher in the CK group compared to all inoculated groups, suggesting a sustained suppressive effect of inoculation on phylogenetic diversity. Simpson and Pielou_e indices showed minor differences among groups, indicating that community evenness was less affected by the inoculation treatment.

#### 2.2.2. Beta Diversity Analysis

**Figure 4:**
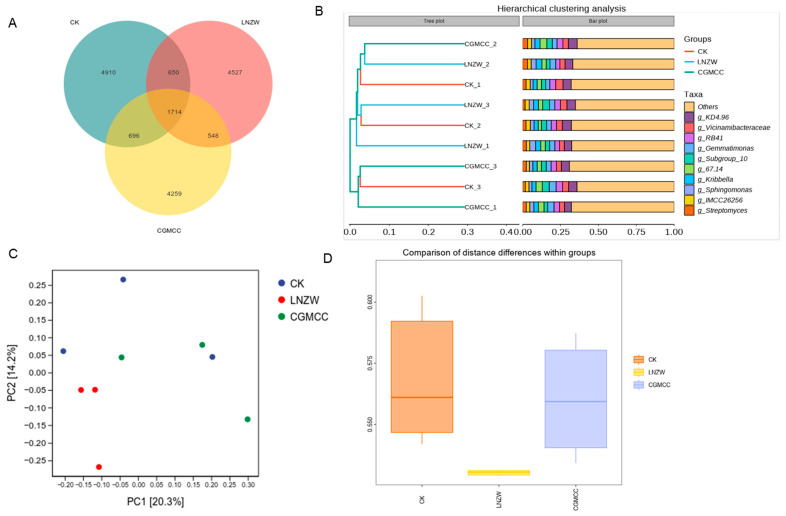
Beta diversity analysis of rhizosphere bacterial communities at 7 days post-inoculation: (**A**) Venn diagram showing shared and unique OTUs among groups. (**B**) Cluster analysis dendrogram. (**C**) Principal Coordinates Analysis (PCoA) plot. (**D**) Bar chart depicting inter-group dissimilarities (beta diversity distance metrics) between the control (CK) and the inoculated groups. Data are presented as mean ± standard deviation.

**Figure 5:**
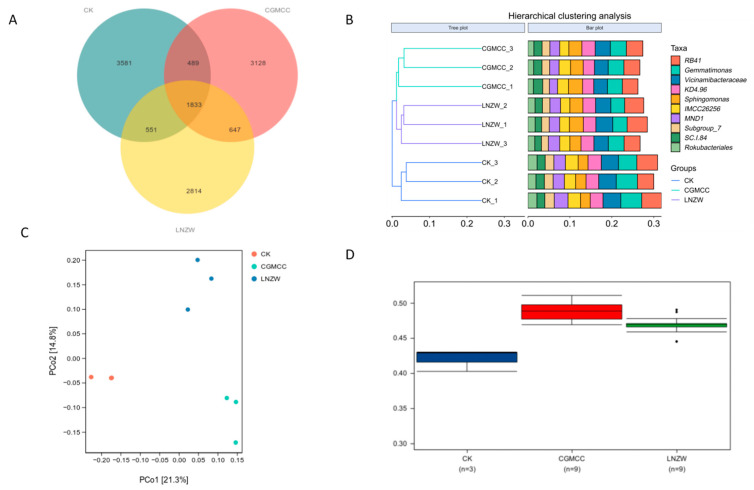
Beta diversity analysis of rhizosphere bacterial communities at 60 days post-inoculation: (**A**) Venn diagram. (**B**) Cluster analysis dendrogram. (**C**) Principal Coordinates Analysis (PCoA) plot. (**D**) Boxplot showing the comparison of intra-group distance differences between the two *A. flavus* strain treatments (LNZW vs. CGMCC).

Venn diagrams (Figure 4A and Figure 5A) revealed the shared and unique operational taxonomic units (OTUs) among the groups. At 7 days post-inoculation (dpi), the control (CK), CGMCC, and LNZW groups shared 1714 OTUs. The number of unique OTUs was 4910 for CK, 4527 for LNZW, and 4259 for CGMCC, indicating a species composition ranking of CK > CGMCC > LNZW. By 60 dpi, the number of shared OTUs increased to 1833. The unique OTU counts were 3581 for CK, 3128 for CGMCC, and 2814 for LNZW, maintaining the trend of CK > CGMCC > LNZW. This suggests a succession in microbial community structure in response to both inoculation treatment and time.

Cluster analysis (Figure 4B and Figure 5B) showed that at 7 dpi, samples from different treatment groups clustered relatively closely. However, by 60 dpi, samples from the inoculated groups (LNZW and CGMCC) were distinctly separated from those of the control group, indicating that the influence of *A. flavus* on root-associated microbial structure became more pronounced over time.

Principal Coordinates Analysis (PCoA) further confirmed this trend (Figure 4C and Figure 5C). The first two principal coordinates (PC1 and PC2) explained 20.3% and 14.2% of the total variation in community structure, respectively. With increasing infection time, the sample points of the infected groups progressively separated from those of the control group. By 60 dpi, they were distributed in distinct regions, demonstrating that *A. flavus* infection significantly altered the structure of the root microbial community.

Analysis of beta diversity distance metrics assessed differences in microbial community structure between groups, highlighting the significant impact of *A. flavus* inoculation. Comparison of intra-group distances revealed a statistically significant distinction between the microbial communities associated with the two different *A. flavus* strains (LNZW vs. CGMCC) (*p* * = 0.016) (Figure 5D). Furthermore, analysis of inter-group dissimilarities (Figure 4D) showed that the rhizosphere microbial communities of the non-inoculated control (CK) group were distinct from those of both inoculated groups (CGMCC and LNZW). The degree of structural differentiation, measured by beta diversity distance metrics, was markedly greater between the control and the inoculated groups than the differences observed between the two inoculated strains themselves.

### 2.3. Composition and Relative Abundance Changes in Dominant Microbial Taxa

**Figure 6:**
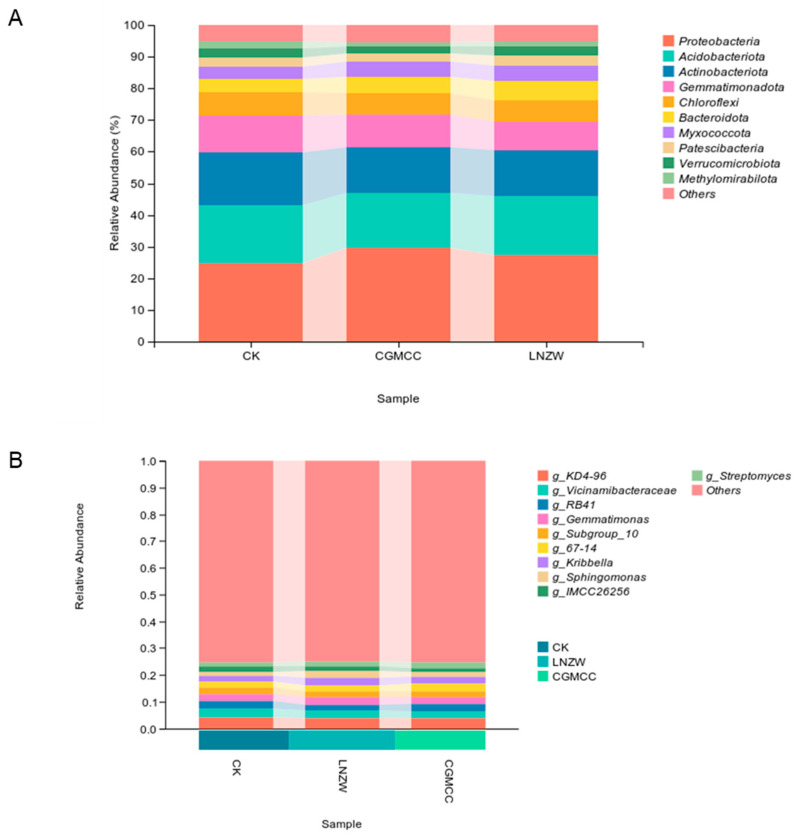
Changes in microbial communities at the (**A**) phylum and (**B**) genus levels at 7 days post-inoculation.

Analysis at the phylum and genus levels revealed significant effects of *A. flavus* inoculation on the bacterial community structure in the peanut rhizosphere at different time points (Figure 6). Stacked bar plots clearly displayed the differences in the relative abundances of the major bacterial phyla between the control group (CK) and the two inoculation treatment groups (CGMCC and LNZW).

A total of ten dominant phyla were identified across all treatments, including Actinobacteria, Proteobacteria, Acidobacteriota, Chloroflexi, Gemmatimonadota, Bacteroidota, Myxococcota, Patescibacteria, Verrucomicrobiota, and Methylomirabilota (Figure 6A). Proteobacteria, Acidobacteriota, and Actinobacteria were the three most dominant phyla, collectively accounting for over 70% of the relative abundance in the CK, CGMCC, and LNZW groups, thereby forming the core of the rhizosphere bacterial community. Other phyla (e.g., Chloroflexi, Bacteroidota) showed relatively balanced proportions, indicating high community diversity.

The relative abundances of the major bacterial genera in the peanut rhizosphere at 7 days post-inoculation (dpi) are shown in Figure 6B. Stacked bar plots illustrate the compositional differences between the control group (CK) and the two *A. flavus* inoculation groups (CGMCC and LNZW). At 7 dpi, the bacterial community at the genus level was primarily composed of several key taxa, including The dominant bacterial taxa included *RB41* (phylum Acidobacteriota, class Acidobacteriae, order *RB41*), *Gemmatimonas*, *Vicinamibacteraceae*, *KD4-96* (phylum Actinobacteriota, class Thermoleophilia, order Gaiellales, family *KD4-96*), *Sphingomonas*, *IMCC26256* (phylum Proteobacteria, class Alphaproteobacteria, order Rhodospirillales, family *IMCC26256*), *MND1* (phylum Nitrospirota, class Nitrospiria, order Nitrospirales, family Nitrospiraceae, genus *MND1*), *Subgroup_7* (phylum Acidobacteriota, class Acidobacteriae, order Acidobacteriales, family Acidobacteriaceae, genus *Subgroup_7*), and *SC-I-84* (phylum Actinobacteriota, class Thermoleophilia, order Solirubrobacterales, family 67–14, genus *SC-I-84*) and *Rokubacteriales*. The “Others” category, representing low-abundance taxa, accounted for a relatively high proportion, reflecting a richness of rare taxa. Notably, compared to the control group (CK), the relative abundances of three genera, *KD4-96*, *Vicinamibacteraceae*, and *RB41*, showed a clear decreasing trend in both inoculation groups (CGMCC and LNZW). In contrast, the genus *Sphingomonas* exhibited a different response pattern, with its relative abundance showing a slight increase or remaining stable in the inoculation groups compared to the control. Other genera, such as *Gemmatimonas* and *IMCC26256*, also displayed variations among groups, although the magnitude of change was less pronounced than in the more responsive taxa mentioned above.

**Figure 7:**
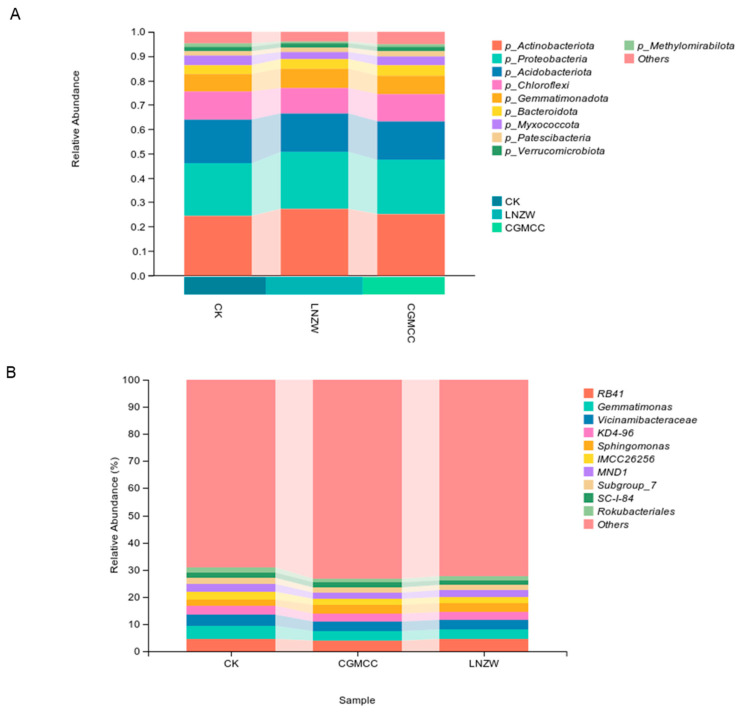
Changes in microbial communities at the (**A**) phylum and (**B**) genus levels at 60 days post-inoculation.

At 60 days post-inoculation (Figure 7), the microbial community composition showed significant changes at both the phylum and genus levels. At the phylum level (Figure 7A), the ten major phyla identified included Actinobacteria, Proteobacteria, Acidobacteriota, Chloroflexi, Gemmatimonadota, Bacteroidota, Myxococcota, Patescibacteria, Verrucomicrobiota, and Methylomirabilota. Among these, some phyla were sensitive to the external intervention. For instance, the relative abundance of Actinobacteria increased from 24.3% in the CK group to 25.0% in the CGMCC group and 27.3% in the LNZW group. The relative abundance of Proteobacteria also rose from 21.8% in the CK group to 22.2% in CGMCC and 23.4% in LNZW, further consolidating its dominance. Meanwhile, the proportions of groups like Acidobacteriota and Chloroflexi were reduced, indicating a shift in the community towards fewer dominant taxa (increased dominance). The bacterial composition also showed clearer differences between the two inoculation treatment groups (CGMCC and LNZW), reflecting strain-specific effects.

At the genus level (Figure 7B), the major genera included *KD4-96*, *Vicinamibacteraceae*, *RB41*, *Gemmatimonas*, *Subgroup_10*, *67–14*, *Kribbella*, *Sphingomonas*, *IMCC26256*, and *Streptomyces*. By 60 dpi, the response trends of several genera to the infection were either sustained or intensified. There was a “specific enhancement” of dominant genera—the proportions of some taxa (e.g., *KD4-96*, *Vicinamibacteraceae*) increased, while the “Others” category decreased substantially, indicating a succession of the community towards a few dominant genera.

## 3. Discussion

### 3.1. Temporal Shifts in Peanut Rhizosphere Bacterial Diversity and Community Structure Induced by Aspergillus flavus Infection

This study uncovered a time-dependent disruption of the peanut rhizosphere bacterial microbiome by *A. flavus* infection, with ecological perturbations emerging prior to visible plant growth inhibition. At 7 days post-inoculation (dpi), no significant differences in peanut plant height were observed between inoculated and control groups, yet bacterial species richness (Chao1, Observed species) and phylogenetic diversity (Faith_pd) were already reduced in the rhizosphere of inoculated plants, and the number of unique operational taxonomic units (OTUs) was markedly lower in the *A. flavus*-treated groups (LNZW < CGMCC < CK). This early decline in microbial diversity indicates that *A. flavus* exerts immediate selective pressure on the rhizosphere bacterial community upon colonization, a phenomenon driven by resource competition and microenvironmental alterations in the rhizosphere niche [27]. This finding aligns with reports in various plant-pathogen systems where “pathogen infection leads to an early decline in rhizosphere microbial diversity” [28]. Notably, similar temporal patterns of early microbial diversity loss have been documented in *A. flavus*-infected maize and cotton rhizospheres [29,30], suggesting that this early microbiome response is a conserved feature across host crops susceptible to *A. flavus*.

By 60 dpi, the restructuring effect of *A. flavus* on the rhizosphere bacterial community became highly pronounced. Beta diversity analysis (PCoA) revealed a clear separation of microbial communities between inoculated and control groups, and intra-group distance analysis confirmed a statistically significant divergence between the two *A. flavus* strains (LNZW vs. CGMCC, *p* = 0.016). This progressive community differentiation demonstrates that *A. flavus* acts not only as a biological filter reducing microbial richness but also as a key driver of rhizosphere community assembly [29,30]. The strain-specific effects on microbiome structure highlight that the ecological impact of *A. flavus* is dependent on the genetic and physiological traits of the infecting strain, which may be linked to differences in toxin production, colonization rate, or metabolic activity between LNZW (a natural toxigenic strain) and CGMCC (a model strain). Notably, the LNZW group exhibited a slight recovery in microbial richness at 60 dpi compared to CGMCC, suggesting potential adaptive changes in the rhizosphere community in response to long-term infection by the natural toxigenic strain.

The reduction in phylogenetic diversity (Faith_pd) persisted throughout the experimental period in inoculated groups, indicating that *A. flavus* infection not only reduces bacterial species number but also disrupts the evolutionary relatedness of the rhizosphere community [31]. This loss of phylogenetic diversity may impair the functional redundancy of the microbiome, as closely related taxa often share similar ecological functions. In contrast, community evenness (Simpson, Pielou_e indices) showed minor changes across groups, implying that *A. flavus* infection primarily affects the composition of the rhizosphere community rather than the relative abundance distribution of dominant taxa [32].

### 3.2. Response of Key Rhizosphere Bacterial Taxa to A. flavus Infection and Their Ecological Implications

At the phylum level, Proteobacteria, Acidobacteriota, and Actinobacteria constituted the core of the peanut rhizosphere bacterial community, collectively accounting for over 70% of the total relative abundance across all treatments. *A. flavus* infection induced a consistent enrichment of Proteobacteria and Actinobacteria at 60 dpi, while reducing the relative abundance of Acidobacteriota and Chloroflexi—an ecological shift closely linked to the stress adaptability of these phyla. Proteobacteria, as typical copiotrophic bacteria, exhibit rapid growth and metabolic versatility, enabling them to exploit the nutrient-rich rhizosphere microenvironment created by *A. flavus* colonization and plant stress responses [31,32]. The enrichment of Actinobacteria, by contrast, is likely a defensive response of the rhizosphere microbiome: members of this phylum produce a diverse array of antifungal secondary metabolites and hydrolytic enzymes, which may mitigate *A. flavus* proliferation and invasion [33]. In addition, Actinobacteria are highly tolerant to biotic stress, making them dominant taxa in the disturbed rhizosphere environment induced by pathogen infection [34].

The decline in Acidobacteriota and Chloroflexi abundance in inoculated groups reflects a shift from a stable, oligotrophic rhizosphere ecosystem to a stress-adapted one [35]. Acidobacteriota are key taxa involved in soil organic matter decomposition and nutrient cycling in low-nutrient environments, and their reduction may impair the rhizosphere’s capacity for nutrient mobilization [36]. Chloroflexi, a phylum associated with microenvironmental stability, plays an important role in maintaining the structural integrity of the soil microbial community; their decline further exacerbates the dysbiosis of the rhizosphere ecosystem [37].

At the genus level, *A. flavus* infection induced distinct responses in key bacterial taxa, with potential implications for peanut health. The relative abundance of *KD4-96*, *Vicinamibacteraceae*, and *RB41* decreased significantly at 7 dpi, a trend that either persisted or was reversed at 60 dpi. *RB41*, a subgroup of Acidobacteria, is closely involved in soil nitrogen and phosphorus cycling [36], and its early decline may reduce the nutrient supply capacity of the rhizosphere, indirectly affecting peanut growth. *Vicinamibacteraceae*, a genus associated with plant growth promotion, plays a role in regulating root development and enhancing plant stress resistance [38]; its reduction may weaken the symbiotic relationship between the peanut and its rhizosphere microbiome. Notably, the relative abundance of these genera increased at 60 dpi, which may represent a compensatory adaptive response of the rhizosphere community to long-term *A. flavus* stress.

In contrast to the above taxa, *Sphingomonas* maintained a relatively stable abundance throughout the infection period, a finding with notable ecological significance. *Sphingomonas* is known for its ability to degrade aromatic compounds and mitigate biotic and abiotic stress in plants [39]; its stability in the infected rhizosphere suggests that this genus may be a core stress-tolerant taxon in the peanut rhizosphere, potentially playing a role in alleviating *A. flavus*-induced stress and aflatoxin detoxification. The increase in the relative abundance of *Streptomyces* (a genus of Actinobacteria) at 60 dpi further supports the defensive response of the rhizosphere microbiome, as *Streptomyces* is a well-characterized producer of antifungal antibiotics and plant growth-promoting substances [33].

The shift in the rhizosphere community toward fewer dominant genera at 60 dpi (marked by a substantial reduction in the “Others” category) indicates that *A. flavus* infection imposes strong selective pressure on the rhizosphere microbiome, favoring the proliferation of stress-tolerant and competitive taxa [31,32]. While this selective enrichment may enhance the short-term adaptability of the rhizosphere community to *A. flavus* stress, it reduces microbial functional redundancy, making the community more vulnerable to subsequent biotic and abiotic perturbations [8].

### 3.3. Mechanistic Links Between Rhizosphere Microbiome Dysbiosis and Peanut Growth Inhibition

The significant inhibition of peanut growth (reduced plant height, shoot and root biomass) at 60 dpi coincided with the structural and compositional dysbiosis of the rhizosphere bacterial microbiome, establishing a clear link between pathogen-induced microbiome changes and plant growth impairment. *A. flavus* infection inhibits peanut growth through a dual mechanism of direct pathogenicity and indirect microbiome-mediated effects. Directly, *A. flavus* produces aflatoxins and phytotoxic metabolites that damage plant cells, inhibit photosynthesis, and disrupt root nutrient absorption [33]. Indirectly, the dysbiosis of the rhizosphere microbiome impairs key ecological functions critical for peanut growth and health, amplifying the inhibitory effects of direct infection.

First, the decline in plant-growth-promoting rhizobacteria (PGPR) such as *Bacillus* and *Pseudomonas* in inoculated groups weakens the rhizosphere’s capacity for plant growth promotion [40]. PGPR enhances plant growth through the secretion of phytohormones (e.g., indole-3-acetic acid), solubilization of insoluble nutrients (e.g., phosphorus, potassium), and biological nitrogen fixation [40]; the reduction in these taxa directly limits peanut nutrient acquisition and growth regulation. Second, the disruption of the rhizosphere microbiome impairs the plant’s induced systemic resistance (ISR) [8]. A stable rhizosphere microbiome is critical for triggering plant ISR against pathogen invasion, and the loss of beneficial taxa reduces the plant’s ability to mount an effective defense response to *A. flavus*, leading to increased pathogen colonization and spread [27,28]. Third, the decline in nutrient-cycling taxa (e.g., *RB41*, Acidobacteriota) impairs the rhizosphere’s capacity for organic matter decomposition and nutrient mobilization, leading to insufficient supply of nitrogen, phosphorus, and other essential nutrients for peanut growth [36,37]. This nutrient limitation further exacerbates the growth inhibition caused by direct *A. flavus* infection.

Furthermore, the structural dysbiosis of the rhizosphere microbiome disrupts microbial network interactions, reducing the community’s “immune” capacity to resist pathogen invasion [41]. In healthy rhizosphere ecosystems, complex microbial interactions form a stable network that inhibits the proliferation of pathogenic microbes through niche competition and microbial antagonism; *A. flavus* infection breaks this network, creating ecological niches for further pathogen colonization and leading to a vicious cycle of microbiome dysbiosis and plant growth inhibition [8]. The strain-specific differences in peanut growth inhibition (LNZW > CGMCC) are consistent with the degree of microbiome dysbiosis induced by the two strains, further confirming that microbiome alterations are a key mediator of *A. flavus*-induced growth suppression.

### 3.4. Limitations of the Study

This study provides novel insights into the impact of *A. flavus* infection on the peanut rhizosphere bacterial microbiome, but several limitations should be acknowledged, which point to directions for future research:

Experimental scale and conditions: The study was conducted under controlled pot experiment conditions with sterilized soil, which cannot fully simulate the complex biotic and abiotic interactions in natural field soils (e.g., indigenous microbial communities, soil texture, climate factors, and crop rotation systems). The sterilization of soil eliminates the native microbiome, which may alter the response of the rhizosphere community to *A. flavus* infection and the plant-microbe-pathogen interaction dynamics [8,27]. Field validation trials under natural soil conditions are therefore necessary to verify the findings of this study.

Focus on bacterial communities only: The study exclusively analyzed the rhizosphere bacterial community and did not investigate other key components of the rhizosphere microbiome, including fungi, archaea, and protists. Fungi (e.g., mycorrhizal fungi) and archaea play important roles in rhizosphere nutrient cycling and plant stress resistance, and their interactions with bacteria and *A. flavus* may shape the rhizosphere ecosystem response to infection. In addition, *A. flavus* itself is a fungal pathogen, and its interaction with the rhizosphere fungal community may be a key driver of microbiome restructuring. Recent studies have shown that manipulating bacterial quorum sensing can significantly shape the structure of the citrus mycobiome, highlighting the importance of investigating interkingdom interactions in the peanut rhizosphere under *A. flavus* stress [42].

Lack of functional and metabolic analysis: The study characterized the rhizosphere bacterial community based on 16S rRNA gene sequencing, which provides information on community composition and diversity but limited insights into microbial functional activity and metabolic potential. *A. flavus* infection likely alters the expression of functional genes related to nutrient cycling, stress response, and antimicrobial activity in the rhizosphere microbiome [27,33]. Integrating multi-omics technologies (metagenomics, metatranscriptomics, metabolomics) is necessary to elucidate the functional responses and metabolic interaction networks of the rhizosphere microbiome under *A. flavus* infection. For example, multi-omics approaches have successfully revealed how beneficial microorganisms like Penicillium baileys W2 inhibit *A. flavus* and promote peanut growth by disrupting membrane permeability and altering the rhizosphere microbial community structure [43]. Similarly, integrated transcriptomics, metabolomics, and microbiomics have been used to uncover plant defense mechanisms against soil-borne pathogens [44].

No analysis of aflatoxin content and microbial detoxification: The study focused on plant growth and microbiome changes but did not quantify aflatoxin content in the rhizosphere soil or peanut tissues, nor did it investigate the aflatoxin detoxification capacity of the rhizosphere microbiome. Some rhizosphere bacteria (e.g., *Sphingomonas*, *Streptomyces*) are known to degrade aflatoxins [34,39], and their activity in the infected rhizosphere may be a key factor in mitigating aflatoxin contamination and plant stress. Recent research has isolated novel aflatoxin-degrading bacteria from peanut rhizospheric soil, such as *Pseudomonas qingdaonensis* [45], and demonstrated that various *Pseudomonas* isolates can effectively control *A. flavus* and aflatoxin B1 production through different enzymatic or non-enzymatic mechanisms [46]. Furthermore, *Achromobacter xylosoxidans* from the peanut rhizosphere has been shown to inhibit *A. flavus* development and aflatoxin synthesis by inducing apoptosis through targeting the cell membrane [47].

Short experimental time scale: The study sampled only at 7 and 60 dpi, providing a snapshot of microbiome changes during the peanut growth period. A more frequent sampling regime (e.g., 14, 28, 42 dpi) would enable a more detailed characterization of the dynamic trajectory of rhizosphere microbiome restructuring in response to *A. flavus* infection, and help identify critical time points for microbiome intervention.

No investigation of microbial inoculation as a mitigation strategy: The study established a link between *A. flavus*-induced microbiome dysbiosis and peanut growth inhibition, but did not test potential mitigation strategies (e.g., inoculation with beneficial rhizobacteria or synthetic microbial communities). Given the role of PGPR and antifungal taxa in plant growth promotion and disease resistance [40,41], testing the efficacy of microbial inoculation to restore the rhizosphere microbiome and alleviate *A. flavus* infection is a critical next step. Research in other crops has demonstrated the effectiveness of synthetic microbial communities (SynComs) in mitigating soil-borne diseases. For instance, SynComs have been shown to rescue strawberries from soil-borne diseases by enhancing soil functional microbial abundance and multifunctionality [48], and well-designed SynComs have proven effective in controlling fire blight disease in apples [49]. Additionally, studies on plant growth-promoting bacteria have revealed complex interkingdom signaling pathways, such as the “root ROS-microbial IAA-root DNA methylation” pathway, that mediate plant-microbe interactions under stress conditions [50].

### 3.5. Future Research Directions

Based on the findings and limitations of this study, future research should focus on the following directions:

Conduct field experiments under natural soil conditions to verify the time-dependent and strain-specific effects of *A. flavus* on the peanut rhizosphere microbiome, and investigate the influence of environmental factors (e.g., soil type, temperature, humidity) on these effects.

Characterize the entire rhizosphere microbiome (bacteria, fungi, archaea, protists) using high-throughput sequencing technologies, and analyze the interkingdom interactions between these microbial groups and *A. flavus* to elucidate the holistic response of the rhizosphere ecosystem to infection.

Integrate multi-omics approaches to investigate the functional and metabolic changes in the rhizosphere microbiome under *A. flavus* infection, and identify key functional genes and metabolic pathways associated with aflatoxin detoxification, plant growth promotion, and disease resistance.

Quantify aflatoxin content in the rhizosphere and peanut tissues, and screen for aflatoxin-degrading rhizosphere bacteria with high activity; investigate the potential of these bacteria for biocontrol of *A. flavus* and aflatoxin contamination.

Construct synthetic microbial communities (SynComs) based on core stress-tolerant and beneficial taxa (e.g., *Sphingomonas*, *Streptomyces*, PGPR), and test their efficacy in restoring the rhizosphere microbiome, enhancing peanut resistance to *A. flavus*, and reducing aflatoxin contamination.

Investigate the interaction between peanut genotypes and the rhizosphere microbiome in response to *A. flavus* infection, and breed peanut varieties with the ability to recruit beneficial rhizobacteria and form a disease-resistant microbiome.

## 4. Conclusions

This study demonstrates that infection by the aflatoxigenic fungus *A. flavus* significantly impairs peanut plant growth and substantially restructures the associated rhizosphere bacterial community. Key findings reveal that while no growth inhibition was observed at 7 days post-inoculation (dpi), a pronounced reduction in plant height, shoot biomass, and root biomass occurred by 60 dpi. Concurrently, high-throughput sequencing analysis showed a clear reduction in bacterial alpha diversity (species richness and phylogenetic diversity) in the rhizosphere of infected plants, particularly at the early infection stage. Beta diversity analysis confirmed a significant and progressive divergence in microbial community structure between infected and control plants over time, with distinct effects also noted between the two *A. flavus* strains used.

At the compositional level, infection enriched for bacterial phyla such as Proteobacteria and Actinobacteria, while reducing the relative abundance of others like Acidobacteriota. Specific genera, including some putative beneficial taxa, were suppressed. These collective alterations suggest that *A. flavus* influences plant health through a dual mechanism: direct pathogenic effects and indirect modulation of the root-associated microbiome, potentially disrupting its supportive functions.

In summary, our work establishes a tangible link between *A. flavus* infection, peanut growth suppression, and rhizosphere microbiome dysbiosis. This insight underscores the importance of microbial community stability in plant-pathogen interactions and provides a foundational framework for developing microbiome-based strategies to enhance crop resilience and mitigate aflatoxin contamination in agroecosystems.

## 5. Materials and Methods

### 5.1. Experimental Materials

Peanut variety selection: The peanut (*Arachis hypogaea* L.) cultivar ‘Baisha’ was provided by the Peanut Research Institute, Liaoning Academy of Agricultural Sciences, Shenyang, China. This variety is characterized by strong growth vigor, broad adaptability, and stable yield, and its response to *A. flavus* infection is considered typical.

*Aspergillus flavus* strain CGMCC 3.4408 was obtained from the China General Microbiological Culture Collection Center (CGMCC).

*Aspergillus flavus* strain LNZW 23 was isolated from peanut rhizosphere soil in Zhangwu County, Liaoning Province, China, by the Oil Crops Research Institute, Chinese Academy of Agricultural Sciences. LNZW 23 is a laboratory code with no public culture collection number.

Culture medium: Potato Dextrose Agar (PDA) was employed for the activation, cultivation, and preservation of *A. flavus*. The medium was prepared according to the following formulation: 200 g of potatoes, 20 g of glucose, 15–20 g of agar, and 1000 mL of distilled water. For preparation, potatoes were peeled, diced, and boiled in distilled water for 30 min. The resulting infusion was then filtered through gauze to obtain the potato extract. Glucose and agar (Both purchased from China National Pharmaceutical Group Chemical Reagent Co., Ltd., Shanghai, China) were subsequently added to the filtrate, and the mixture was heated with continuous stirring until all components were completely dissolved. The medium was then dispensed into appropriate vessels and sterilized by autoclaving at 121 °C for 20 min.

Reagents: The OMEGA E.Z.N.A. Soil DNA Kit (Bao Bioengineering Co., Ltd., Dalian, China) was used for total microbial DNA extraction from peanut rhizosphere soil. The PCR primers used in this study were synthesized by Shenggong Biotechnology Co., Ltd. (Shanghai, China). Universal primers 27F (5′-AGAGTTTGATCMTGGCTCAG-3′) and 1492R (5′-TGGYTACCTTGTTACGACTT-3′) were employed to amplify the nearly full-length bacterial 16S rRNA gene (V1–V9 regions). Additionally, routine reagents including absolute ethanol, chloroform, isoamyl alcohol, Tris-HCl, and EDTA (all analytical grade) were purchased from China National Pharmaceutical Group Chemical Reagent Co., Ltd. (Shanghai, China).

### 5.2. Experimental Design

A controlled pot experiment was conducted in a climate chamber to investigate the effects of *A. flavus* infection on peanut plants. The experiment consisted of three groups: a treatment group (inoculated with two *A. flavus* strains: one group was inoculated with the *A. flavus* strain CGMCC 3.4408, referred to as Group CGMCC, while the other group was inoculated with LNZW 23, abbreviated as Group LNZW) and a control group (mock-inoculated with an equivalent volume of sterile water), each comprising ten biological replicates.

Peanut seeds were surface-sterilized with 75% ethanol (Sinopharm Chemical Reagent Co., Ltd., Shanghai, China) for 1 min, rinsed 2–3 times with sterile distilled water, and placed on moist sterile filter paper for germination in an incubator at 28 °C for 2–3 days. On May 20, uniformly germinated seeds with emerged radicles were selected and sown in pots (two seeds per pot). Each pot (15 cm diameter) was filled with 1.5 kg of field soil (loam; pH 6.8; organic matter content 18 g/kg), which had been sterilized by autoclaving at 121 °C for 2 h. After emergence, seedlings were thinned to one robust plant per pot (Figure S1). The pots were maintained in a climate chamber under natural light and temperature conditions, with regular and quantitative watering to ensure consistent soil moisture throughout the experiment.

At the pegging stage (15 July), plants in the treatment group were inoculated by injecting an *A. flavus* spore suspension into the rhizosphere soil. To prepare the inoculum, preserved *A. flavus* strains were cultured on potato dextrose agar (PDA) plates at 28 °C for 7–10 days until abundant conidial production. Conidia were harvested by rinsing the colony surface with sterile distilled water, and the spore suspension was adjusted to a concentration of 1 × 10^7^ spores/mL. Using a sterile syringe, 10 mL of this suspension was injected into the rhizosphere soil of each treatment plant. Control plants received an equal volume of sterile water injected in the same manner.

Throughout the experimental period, plants were maintained under natural cultivation conditions with regular and quantitative watering to maintain uniform soil moisture.

### 5.3. Sample Collection and Processing

Samples were taken at 7 and 60 days (dpi) after vaccination. Collect rhizosphere soil using the shaking method. Specifically, use sterile scissors to remove the aboveground part of the peanut plant, and then gently remove the entire root system and attached soil from the flowerpot. After discarding loose and attached large soil blocks, carefully brush off the soil tightly attached to the root surface (defined as the 0–4 mm area) with a sterile brush and collect it as rhizosphere soil. The rhizosphere soil of each plant is considered an independent biological replica, placed in a separate sterile self-sealing bag, and immediately transported to the laboratory on ice. Fresh rhizosphere soil samples were rapidly stored at −80 °C for subsequent soil microbial metagenomic DNA extraction and high-throughput sequencing analysis.

### 5.4. Microbial Diversity Analysis

High-throughput sequencing was employed to analyze the diversity of the peanut root-associated microbiome. First, total microbial DNA was extracted from rhizosphere soil samples using the OMEGA E.Z.N.A. Soil DNA Kit according to the manufacturer’s instructions. DNA integrity was verified by 1% agarose gel electrophoresis, and concentration/purity were determined using a NanoDrop 2000 spectrophotometer (Changzhou Meikuanda Electronic Appliance Sales Co., Ltd., Changzhou, China) to ensure quality for downstream applications.

Using the extracted DNA as a template, PCR amplification was performed with the aforementioned primers targeting the bacterial 16S rRNA gene. The PCR reaction mixture (25 μL) contained: 12.5 μL of 2× Taq PCR Master Mix, 0.5 μL each of forward and reverse primers (10 μmol/L), 1 μL of DNA template, and 10.5 μL of ddH_2_O. The PCR conditions were: initial denaturation at 95 °C for 5 min; 35 cycles of 95 °C for 30 s, 55 °C for 30 s, and 72 °C for 30 s; final extension at 72 °C for 10 min.

PCR products were examined by agarose gel electrophoresis. Target bands were excised and purified using the Axygen DNA Gel Extraction Kit (Corning, Shanghai, China). The purified amplicons were subjected to high-throughput sequencing on an Illumina MiSeq platform, performed by Nanjing Paisennuo Biotechnology Co., Ltd. (Nanjing, China).

Raw sequencing data underwent quality control to remove low-quality reads, adapter sequences, and chimeras. High-quality sequences were clustered into operational taxonomic units (OTUs) at a 97% similarity threshold using Quantitative Insights Into Microbial Ecology (QIIME, v2022.11, QIIME 2 Development Team, Boulder, CO, USA) software. Taxonomic annotation of each OTU was performed by comparison against the Ribosomal Database Project (RDP, v11.4, Michigan State University, Michigan, USA) database to determine microbial identities.

Microbial diversity indices, including the Shannon index, Simpson index, Ace index, and Chao1 index, were calculated. The Shannon and Simpson indices assess community diversity (higher values indicate greater diversity), while the Ace and Chao1 indices estimate species richness (higher values indicate greater richness). Community structure differences among treatment and control groups were visualized using Principal Component Analysis (PCA), Principal Coordinates Analysis (PCoA), and Non-metric Multidimensional Scaling (NMDS). Graphs were generated using OriginPro (v2023b, OriginLab Corporation, Northampton, MA, USA).

### 5.5. Statistical Analysis

Experimental data were initially collated and summarized using Microsoft Excel 2019 (16.0.10356.20006) to calculate means and standard deviations. Significance testing was performed using Excel 2019 software. Differences in microbial diversity indices, richness indices, and relative abundances of microbial taxa between treatment and control groups were compared using one-way analysis of variance (ANOVA). Differences were considered statistically significant at *p* < 0.05.

## Data Availability

The raw sequence data reported in this paper have been deposited in the Genome Sequence Archive (Genomics, Proteomics & Bioinformatics 2025) in the National Genomics Data Center (Nucleic Acids Res 2025), China National Center for Bioinformation/Beijing Institute of Genomics, Chinese Academy of Sciences (GSA: CRA037308) that are publicly accessible at https://ngdc.cncb.ac.cn/gsa, accessed on 31 January 2026.

## Supplementary Materials

The following supporting information can be downloaded at https://www.mdpi.com/article/10.3390/toxins18030131/s1, Table S1: Growth parameters of peanut plants at 7 and 60 days post-inoculation with *A. flavus*. Table S2: Bacterial alpha diversity indices at 7 days post-inoculation with *A. flavus.* Table S3: Bacterial alpha diversity indices at 60 days post-inoculation with *A. flavus*. Figure S1: Peanut pot experiment.
