# Supplementary material for: Impact of Aspergillus flavus Infection on the Rhizosphere Bacterial Microbiota of Peanut (Arachis hypogaea L.)
Source: Toxins (Basel). 2026 Mar 5;18(3):131. doi: 10.3390/toxins18030131 (PMC13030443; doi:10.3390/toxins18030131)
Supplement: Supplementary file 1 [file toxins-18-00131-s001.zip › toxins-4158100-supplementary.pdf]

Table S1. Growth parameters of peanut plants at 7 and 60 days post-inoculation with *A. flavus*.

Sample ID	Plant Height (cm)	Shoot Biomass (g)	Root Biomass (g)	Sample ID	Plant Height (cm)	Shoot Biomass (g)	Root Biomass (g)
CK1-7d	13.3	1.05	0.51	CK1-60d	32.4	3.25	1.55
CK2-7d	13.0	1.03	0.49	CK2-60d	32.1	3.31	1.60
CK3-7d	12.8	1.08	0.53	CK3-60d	32.5	3.22	1.50
CK-7d(average)	13.0	1.05[a]	0.51[a]	CK-60d(average)	32.3[a]	3.26[a]	1.55[a]
LNZW1-7d	13.1	0.98	0.47	LNZW1-60d	26.2	2.30	1.00
LNZW2-7d	12.7	1.00	0.49	LNZW2-60d	25.3	2.26	1.03
LNZW3-7d	12.9	0.95	0.46	LNZW3-60d	24.9	2.34	0.98
LNZW-7d(average)	12.9	0.98[b]	0.47[b]	LNZW-60d(average)	25.5[c]	2.30[c]	1.00[b]
CGMCC1-7d	13.0	1.00	0.48	CGMCC1-60d	26.3	2.40	1.10
CGMCC2-7d	12.8	0.98	0.50	CGMCC2-60d	26.8	2.35	1.05
CGMCC3-7d	12.6	1.03	0.47	CGMCC3-60d	26.2	2.45	1.00
CGMCC-7d(average)	12.8	1.00[ab]	0.48[ab]	CGMCC-60d(average)	26.4[b]	2.40[b]	1.05[b]

Note: abc represents P<0.05 difference significant

Table S2. Bacterial alpha diversity indices at 7 days post-inoculation with *A.flavus.*

Sample	Chao1	coverage	Simpson	Pielou_e	Shannon	Observed_species	Faith_pd
CK_1	3598.04	0.990005	0.998689	0.915799	10.7837	3505	208.716
CK_2	3321.14	0.993750	0.998694	0.917029	10.7116	3283	206.258
CK_3	3319.20	0.994323	0.998786	0.922008	10.7706	3285	213.633
CGMCC_1	3115.47	0.993357	0.998187	0.910725	10.5473	3064	196.542
CGMCC_2	3240.76	0.991787	0.998542	0.915801	10.6493	3166	192.458
CGMCC_3	3066.05	0.994142	0.998475	0.913258	10.5597	3025	192.825
LNZW_1	3317.30	0.992693	0.998449	0.913728	10.6642	3261	197.634
LNZW_2	3184.86	0.993297	0.998281	0.909354	10.5606	3133	193.951
LNZW_3	3259.01	0.993116	0.998580	0.915926	10.6690	3210	192.853

Table S3. Bacterial alpha diversity indices at 60 days post-inoculation with *A. flavus*.

Sample	Chao1	coverage	Simpson	Pielou_e	Shannon	Observed_species	Faith_pd
CK_1	3541.61	0.983652	0.998809	0.909588	10.6452	3334.7	317.342
CK_2	3019.27	0.992245	0.998967	0.928264	10.6998	2950.3	257.185
CK_3	2894.22	0.992278	0.998901	0.926265	10.6163	2819.8	275.111
CGMCC_1	2557.46	0.995712	0.998809	0.925928	10.4679	2530.6	230.564
CGMCC_2	3138.43	0.989740	0.998963	0.925664	10.7019	3022.2	271.786
CGMCC_3	3277.20	0.988853	0.998992	0.926419	10.7639	3145.0	291.494
LNZW_1	2886.04	0.992491	0.998954	0.928619	10.6369	2806.5	234.135
LNZW_2	2842.24	0.993417	0.998932	0.929709	10.6360	2778.6	217.722
LNZW_3	3018.33	0.991056	0.998726	0.920155	10.5954	2926.1	280.427

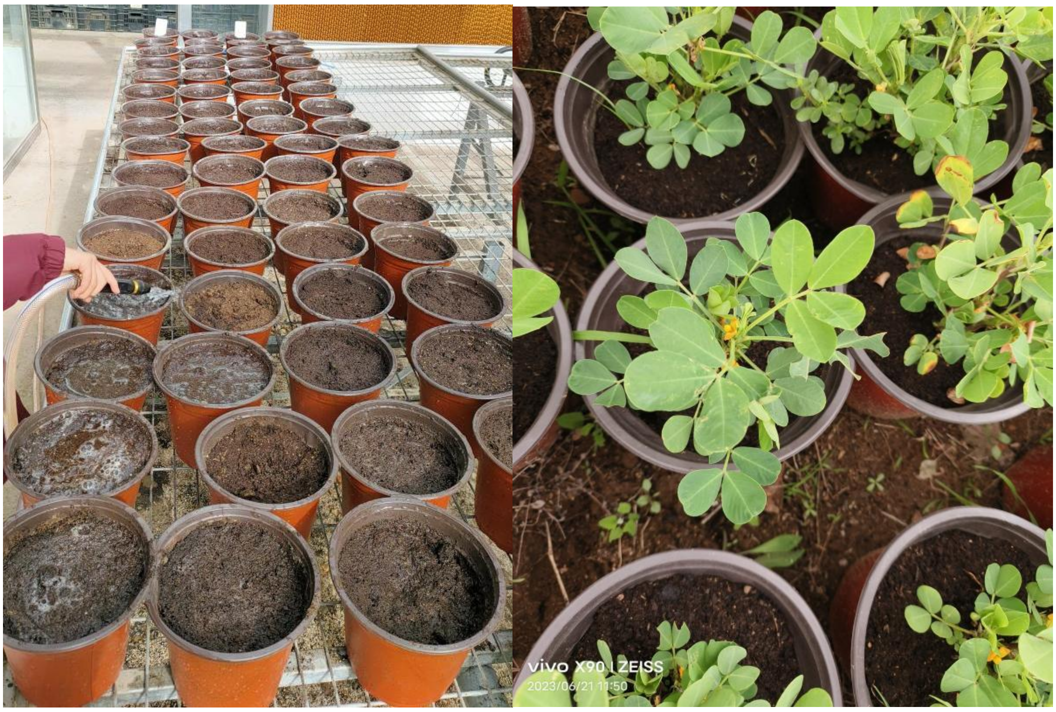

Figure S1. Peanut pot experiment
